# Supplementary material for: Association between executive functions and COMT Val108/158Met polymorphism among healthy younger and older adults: A preliminary study
Source: PLoS One. 2024 May 13;19(5):e0303343. doi: 10.1371/journal.pone.0303343 (PMC11090336; doi:10.1371/journal.pone.0303343)
Supplement: S1 Table — (DOCX) [file pone.0303343.s001.docx]

S1Table. Summary data for executive composite scores

|  |  | Inhibition | Shifting | Updating |
| --- | --- | --- | --- | --- |
| Younger | VV | 0.403 (0.573) ± 0.139 | 0.299 (0.199) ± 0.178 | 0.507 (0.367) ± 0.228 |
|  | VM | 0.211 (0.146) ± 0.225 | 0.219 (0.030) ± 0.251 | 0.099 (-0.149) ± 0.240 |
|  | MM | 0.815 (0.636) ± 0.148 | 0.477 (0.260) ± 0.197 | 0.117 (0.171) ± 0.214 |
| Older | VV | -0.263 (-0.535) ± 0.284 | 0.027 (-0.147) ± 0.263 | -0.398 (-0.360) ± 0.242 |
|  | VM | -0.863 (-0.597) ± 0.247 | -0.265 (-0.325) ± 0.212 | 0.247 (-0.062) ± 0.251 |
|  | MM | -0.256 (-0.291) ± 0.230 | -0.089 (-0.027) ± 0.287 | -0.111 (-0.414) ±0.244 |

Summary data for the Inhibition, Shifting and Updating tasks for the younger and older individuals in the three genotype groups (VV; VM; MM). Median values are followed by Mean values in brackets, and +- Std. Error of Mean.
