# Supplementary material for: Association between executive functions and COMT Val108/158Met polymorphism among healthy younger and older adults: A preliminary study
Source: PLoS One. 2024 May 13;19(5):e0303343. doi: 10.1371/journal.pone.0303343 (PMC11090336; doi:10.1371/journal.pone.0303343)
Supplement: Attachment — Submitted filename: REVIEW-PONE-D-23-21633.docx [file pone.0303343.s002.docx]

**Review PONE-D-23-21633**

**Association between executive functions and COMT Val108/158Met polymorphism among healthy younger and older adults**

**Overview**

The manuscript investigated the association of executive functions and polymorphism in young and older adults. In general, the manuscript is organized. I suggest improving the Figure's quality. For example, in Figure 2, it is difficult to understand the groups.

**Specific comments:**

**Keywords**

The keywords have “COMT and Executive functions”, both in the manuscript's Title. A suggestion for keywords is not to use the same ones already in the title because it increases the chances of your work being found on search engines.

**Introduction**

This session introduces three executive functions (EF), i.e., inhibition, shifting and updating, based on the study of Miyake et al. (2000). This study investigated the contributions of EF to Complex “Frontal Lobe” Tasks. However, in the Introduction, it was defined the PFC area. Other EFs may deteriorate with age, such as planning, reaction time or flexibility. So, please clarify why it was used only these three EFs.

**Method**

The sample was described by age and males in each group (e.g., Younger: 28 Men, M age = 23.47 years old; SD = 3.12; range = 18-30). What about the women’s characteristics? Please describe it in both groups of age.

There is missing information in this session. Let me be clear: In what order were the tests administered? Was the test order controlled between participants? How long did it last? Was there an interval between the tests? Were all the tests administered in a single session or multiple sessions? Please insert all procedures to allow one to replicate the experiment.

Please insert a reference for the classes adopted for effect t size.

Please insert procedures for analyzing data normality before data analyses.

Item **2.6 Statistical analyses** didn't describe the Statistical procedures, but only the software. Please describe all statistical procedures, including data normality and sample size.

**Results**

When an ANOVA has main effects, post hoc is carried out to identify the differences. The item **3.3. Genetic-related effects on executive functioning**, ANOVA didn’t have main interactions, and there is no need to run a post-hoc. Although you can find a few papers that run post-hoc without main interaction, running Bonferroni's post-hoc when the “p” value was high, and effect size low (i.e., p=0.16, ƞ2 =0.07) is quite strange. Following this way of thinking, these results cannot be discussed.

**Discussion**

Since the results of both hypotheses are not considered (please, check the results comments), the discussion should go in the opposite direction and be rewritten.
