# Supplementary material for: Association between executive functions and COMT Val108/158Met polymorphism among healthy younger and older adults: A preliminary study
Source: PLoS One. 2024 May 13;19(5):e0303343. doi: 10.1371/journal.pone.0303343 (PMC11090336; doi:10.1371/journal.pone.0303343)
Supplement: Attachment — Submitted filename: COLLETTE Response to reviewers.docx [file pone.0303343.s003.docx]

**Reviewer #1**

**Comment 1. Overview**
*The manuscript investigated the association of executive functions and polymorphism in young and older adults. In general, the manuscript is organized. I suggest improving the Figure's quality. For example, in Figure 2, it is difficult to understand the groups.*

**Response.** As suggested by Reviewer 1, quality of our two Figures was improved.

**Comment 2. Keywords**
*The keywords have “COMT and Executive functions”, both in the manuscript's Title. A suggestion for keywords is not to use the same ones already in the title because it increases the chances of your work being found on search engines.*

**Response.** Based on the reviewer’s comment, we have changed the keywords accordingly. Keywords are now: Aging, Inhibition, Shifting, Updating, Cognitive control, Genetics (**Page 2**)

**Comment 3. Introduction**
*This session introduces three executive functions (EF), i.e., inhibition, shifting and updating, based on the study of Miyake et al. (2000). This study investigated the contributions of EF to Complex “Frontal Lobe” Tasks. However, in the Introduction, it was defined the PFC area. Other EFs may deteriorate with age, such as planning, reaction time or flexibility. So, please clarify why it was used only these three EFs.*

**Response.** We have slightly reformulated the first two sections of the introduction to state more clearly that we do not consider that only inhibition, shifting and updating are impaired by aging, but that we are interested in these three functions because Miyake et al. (2000) showed they refers to separate cognitive constructs. We have also specified that flexibility is impaired by aging and that flexibility and shifting refers to very similar concepts. Finally, we have also specified that executive functions does not totally rely on frontal lobes.

**Page 3:1:3:** Age related executive difficulties were first observed in complex and multicompound tasks involving planning, conceptualization and problem solving (for a review see (3)). For example, lower performance by comparison to younger people is frequently reported for the three distinct functions of inhibition, shifting and updating of information identified by Miyake et al. (4) as separate cognitive processes. However, shifting and inhibition tasks are not found systematically impaired during aging (for a review Collette & Salmon, 2014). For the shifting process (that also refers to flexibility abilities), the capacity to maintain and manipulate two mental sets simultaneously is more sensitive to the age-effect than the capacity to alternate between the two sets (Verhaeghen & Cerella, 2002; Wasylyshyn et al., 2011 for meta-analyses).

**Page 3:2:1:** Several authors proposed that changes in prefrontal cortex (PFC) structure and functions explain a large part of changes in executive functioning, (for reviews, Dennis & Cabeza, 2008; Park & Reuter-Lorenz, 2009).

**Comment 4. Method**
*(a) The sample was described by age and males in each group (e.g., Younger: 28 Men, M age = 23.47 years old; SD = 3.12; range = 18-30). What about the women’s characteristics? Please describe it in both groups of age.*

**Response.** In the previous version of the manuscript, we reported the number of males in our sample for brevity’s sake. We have now specified the number of males/females. However, as a gender comparison will be not performed, the description of demographic information separately for both groups does not seem useful here.

**Page 6:2:1**. Fifty-five younger (F/M 27/28; M age = 23.47 years old; SD = 3.12; range = 18-30) and 45 older (F/M 26/19; M age = 67.37 years old; SD = 4.9; range = 60-75) subjects were included in the present study.

*(b) There is missing information in this session. Let me be clear: In what order were the tests administered? Was the test order controlled between participants? How long did it last? Was there an interval between the tests? Were all the tests administered in a single session or multiple sessions? Please insert all procedures to allow one to replicate the experiment.*

**Response.** The information about testing session is now added.

**Page 6:2:4.** The testing session lasted between 1h30 and 2h, with short breaks provided between cognitive tasks, when required by the participant.

**Page 7:2:4.** Task administration was in the same order as presented below, and similar for all participants.

**Page 10:3:7.** The Mattis scale was administered before the executive tasks and the tasks assessing intelligence and processing speed were administered at the end of the testing session.

*(c) Please insert a reference for the classes adopted for effect t size.*

**Response.** We do not understand the comment of the reviewer.

*(d) Please insert procedures for analyzing data normality before data analyses.
Item 2.6 Statistical analyses didn't describe the Statistical procedures, but only the software. Please describe all statistical procedures, including data normality and sample size.*

**Response.** A lack of description of our statistical analyses was also mentioned by Reviewer 2. Statistical information was sparsely provided in the results section but we agree with the reviewers that this was not complete and explicit enough. Consequently, our analyses are now detailed at section 2.6. Results of assumptions check for student *t* test were already presented in the previous version of the manuscript. (footnote in the result section, **Page 15:1:1** in the current version), and assumption checks are now added for the ANOVA:

**Page 12:2:2.** Results with p < .05 were considered as statistically significant. Analyses were run on the sample size of 100 participants. Missing data (four for the whole dataset) were replaced by the mean value in the participant group for this variable.

The effect of age on executive functioning was assessed with Student *t* test between younger and older participants. As detailed in the method section, measures were composite Z scores centered on mean and standard deviation of the younger group. To determine if age-related changes in executive functioning is modulated by COMT polymorphism, we performed an analysis of variance (ANOVA) with the 6 groups as an independent variable, and executive scores as an intra-subject measure. The dependent variables were composite Z-scores centered on mean and standard deviation on the whole sample. Bonferroni post-hoc tests were carried out to explore, for each executive process, the presence of difference between younger and older participants from the same genotype or between the three genotypes within the same age group. If another difference was statistically different, it was reported in the result section for sake of completeness only. Data distribution and variance homogeneity for composite scores used in Student *t* test and ANOVA were analyzed with Shapiro-Wilk and Levene tests, respectively.

**Page 15:3:5:** Results of assumptions check showed that the repeated measures ANOVA can be applied because the data did not severely violate the conditions of application. Shapiro-Wilk tests showed a normal distribution of the data [Inhibition W=0.984 p=0.253; Flexibility W=0.988 p=0.528; Updating W=0.976 p=0.061]. Levene’s test indicates that variance are approximatively equal across the six groups [inhibition F(5,94)=1.26, p=0.29; Flexibility F(5,94)=1.47, p=0.21; Updating F(5,94)=0.44, p=0.82]. The Mauchly test revealed that sphericity was not respected χ^2^(5)=12.86, p<0.005). However, the epsilon values of the Greenhouse (0.886) and Huynh-Feldt (0.949) correction being close to 1 suggest a slight violation of sphericity.

*(e) Results. When an ANOVA has main effects, post hoc is carried out to identify the differences. The item 3.3. Genetic-related effects on executive functioning, ANOVA didn’t have main interactions, and there is no need to run a post-hoc. Although you can find a few papers that run post-hoc without main interaction, running Bonferroni's post-hoc when the “p” value was high, and effect size low (i.e., p=0.16, ?2 =0.07) is quite strange. Following this way of thinking, these results cannot be discussed.*

**Response.** We do not agree with this comment. Indeed, with a priori hypotheses, we can look at all the contrasts we consider of interest. However, in the absence of an interaction, we need to be very careful about the conclusions that can be drawn, and the results can't be interpreted as if there was an interaction. We can only say that there is a difference for one group, but not for another, without being able to state that this difference in differences is robust. We agree that our a priori hypotheses were not clearly stated in the previous version of the manuscript. This is now done **Page 12:3:7.** Moreover, in the previous version of the manuscript, it was already stated in the result section that unexpected significant differences are reported only for sake of completeness **(Page 20:2:3** current version) and in our discussion, we refrained from discussing the post-hoc results as interaction effect.

**Page 12:3:7:** Bonferroni post-hoc tests were carried out to explore, for each executive process, the presence of difference between younger and older participants from a same genotype group or between the three genotypes within the same age group. If another comparison showed a significant difference, it was reported in the result section for sake of completeness only.

**Comment 5. Discussion**
*Since the results of both hypotheses are not considered (please, check the results comments), the discussion should go in the opposite direction and be rewritten.*

**Response.** We do not agree with this comment. Indeed, we also considered in the previous version of the manuscript the possibility of an absence of COMT effect due to the age of our participants and other genetic influence not controlled for in this study (see **pages 18:2, 19:3**, and **20:1**, current version of the manuscript, no changes made).

**Reviewer #2**

**Comment 1. Overview**

*Article “Association between executive functions and COMT Val108/158Met polymorphism among healthy younger and older adults“ written by Apa et al. is impressive in the number of used neuropsychological tests. However, the number of subjects involved in this study (overall 100 participants) is too small for serious genetic analysis. Since you have healthy participants, you should include at least 500 participants. Thus, I suggest the rejection of this article.*

**Response:** As indicated in the Cover Letter and the 4.5 section *Strength and Limitations* (previous version of the manuscript), the interest of the study is the complete phenotyping of executive functioning with several measures for each function that allow computing composite scores that are purer measures of executive functioning. In that sense, the number of participants corresponds to the standard in the cognitive psychology research field. This was stated in the previous version of the manuscript (**Page 24:3:3** current version). To take into account comment 1 of Reviewer 2, we have added a sentence indicating that, due to our sample size, these results need replication.

**Page 22:2:6:** Consequently, presence of a lower performance during aging on the composite score of inhibition (but not on shifting or updating scores) for homozygous Val carriers needs replication with protocols targeting for example one specific executive process. This will allow inclusion of a larger number of participants than in this study.

**Comment 2.** **Abstract**
*It should be „in comparison to younger Val/Val.“ Instead of „by comparison to younger Val/Val.“*

**Response:** We have modified the manuscript accordingly (see Page 2).

**Comment 3.** **Methods**

*(a) you should not present age as a mean value, but as median*

**Response.** It is common practice in cognitive aging studies to report age mean along with range. For consistency with other published studies on the same topic, we prefer to keep mean and standard deviation.

*(b) what does the numeric unit „ll“ stand for (you mentioned it in the paragraph 2.2.)
- you should write „°C“ instead of“_C“*

**Response.** We apologize for the typos remaining in that part of the manuscript. “II” stand for “µl” and “_C” for “°C”. We must thank the reviewer for the attentive reading. It allows us to rewrite all the paragraph in a more appropriate way (see **Pages 6-7**).

*(c) Paragraph „2.6. Statistical analyses“ should be written more thoroughly. Authors should explain which exact tests they used in their analysis (not only the statistical programme in which the analysis was performed).*

**Response.** We thank the Reviewer for pointing out the Statistical analyses section issue. A full description has now been added to the revised version of the manuscript. A similar comment was addressed by Reviewer 1 (see Comment 4d and **Page 12:2:2, 15:2:1** and **15:3:5** in the current version of the manuscript).

**Comment 4. Results**

*(a) In section „Methods“ the authors used period as decimal point, while in section „Results“ authors used comma as decimal point. This has to be corrected.*

**Response:** We agree with the reviewer that decimal separator should be used consistently. Hence, the decimal separator has been changed to period throughout the revised version of the manuscript.
*(b) In the „Table 1.“ you should also add the column on the results of statistical analyses.*

**Response:** Results of statistical analyses were indicated in the text before Table 1 (section Demographic data) in the previous version of the manuscript (now Page 13). There will be redundancy if we add it also in Table 1

*(c) You should not mention statistical tests in the „Results“ section, but you should explain which test you used for what kind of analysis in paragraph „2.6. Statistical analyses“.*

**Response:** A similar comment was addressed by Reviewer 1 and we modified the text accordingly (see Comment 4d and Page 12:3:1). However, we kept in footnotes (Page 15 and 17) rationale for confirmatory supplementary statistical analyses when normal distribution or variance homogeneity is not present.

*(d) In the „Table 2.“ you should also add the column on the results of statistical analyses (comparison of variables between the groups).*

**Response:** Results of statistical analyses are now added. For consistency with the comparison between younger and older participants, the results are included in the main text.

**Page 13:2:4:** ANOVAs on educational level [F(2,52)=0.22], fluid intelligence [F(2,52)=0.89], vocabulary level [F(2,52)=2.72] and speed of processing [F(2,52)=1.75] did not show significant between-group differences in younger participants (all p>0.05). For older adults, a lower performance for the processing speed task was observed for Val/Met participants [F(2,42)=5; p=0.01, ƞ² =0.19, Bonferroni post-hoc test] while there is no significant group differences (all p>0.05) for educational level [F(2,42)=0.63], fluid intelligence [F(2,42)=1.56], vocabulary level [F(2,42)=1.62] and score at the Mattis dementia rating scale [F(2,42)=1.04].

*(e) The resolution of figure 2. is poor and it should be improved.*

**Response.** The resolution of Figure 2 (as well as Figure 1) was improved. Please note that we lost resolution at the previous submission due to uploading guidelines.  **Comment 5. Discussion**

*(a) You should mention if any GWAS studies were conducted before by using the results of cognitive tests you used in your study as quantitative traits.*

**Response:** GWAs studies are now included in the discussion. We capitalized on this comment to also add two recent publications in the discussion.

**Page 18:2:1:** At this time, few genome-wide association studies (GWAs) were conducted to assess the potential genetic contribution to executive functioning. Dueker et al (81) observed that a composite measure of executive function is associated with four SNPs located in the long intergenic non-protein coding RNA 1362 gene, LINC01362, on chromosome 1. The associated SNPs have been shown to influence expression of the tubulin tyrosine ligase like 7 gene, TTLL7 and the protein kinase CAMP-activated catalytic subunit beta gene, PRKACB, in several regions of the brain involved in executive function. Hatoum et al. (82) observed that genes related to GABAergic pathways influence executive functions (assessed by a common factor) while the effect of COMT Val/Met polymorphism was not significant at the genome-wide level.

**Page 20:2:17:** Finally, an interactive effect was also observed for both COMT and APOE for semantic fluency, with Val/Met heterozygous individuals performing better only in the context of an ApoE Ɛ4 allele (95).

**Page 21:2:16:** A similar interpretation was proposed by de Frias et al. (97) to explain differences in pattern of brain activity during an *n-back* task: increased transient activity in the medial temporal lobe was observed in the Met/Met group and interpreted as a decrease of updating abilities while increased sustained activity in the prefrontal cortex was reported for the Val/Val group and attributed to less efficient maintenance processes (see also (98,101)).

*(b) instead of „nucleotide polymorphism“ should be „single nucleotide polymorphism“*

**Response:** We thank the Reviewer for this specification. The revised version of the manuscript reflects this change.

**Comment 6. References.**

*References are poorly written, sometimes you write them as Vancouver style, and sometimes as Harvard style.*

**Response.** We thank the Reviewer for pointing out the lack of references style consistency. All references were converted to Vancouver style.
