# Supplementary material for: Association between executive functions and COMT Val108/158Met polymorphism among healthy younger and older adults: A preliminary study
Source: PLoS One. 2024 May 13;19(5):e0303343. doi: 10.1371/journal.pone.0303343 (PMC11090336; doi:10.1371/journal.pone.0303343)
Supplement: Attachment — Submitted filename: ResponseToReviewers_R2.docx [file pone.0303343.s004.docx]

**Response to comments of reviewers.**

We are sincerely grateful for the reviewing process. Please find enclosed the new version of the manuscript previously entitled **“Association between executive functions and COMT Val^108/158^Met polymorphism among healthy younger and older adults”**, which we have the pleasure to resubmit for publication in *Plos One.* We are thankful to the Reviewers for their examination of our manuscript and for providing comments that helped to improve its content.

You will find a point-by-point reply to the topics raised by the Reviewers along with a mention of the pages and paragraph containing the changes (underlined in yellow) carried out in the new version of the manuscript.

**Reviewer #2:**

**Comment 1**. Since the authors could not include at least 500 participants in their genetic study, as I requested, they should change the article's title. The title should be; „Association between executive functions and COMT Val108/158Met polymorphism among healthy younger and older adults; a preliminary study“.

**Response.** This was done as requested by the reviewer #2, see **Title Page 1**: “Association between executive functions and COMT Val108/158Met polymorphism among healthy younger and older adults : a preliminary study”

**Comment 2.** I do not agree with the authors that the practice is to present data on age as a mean value, and I think it should be presented as a median. This is in fact, a common statistical error, and you have to correct this.

**Response.** In a review of research methods, Pickering (2017) discussed how to present descriptive statistics related to participants. According to this paper, it is prescribed to report age as mean, except in case of a skewed distribution. Regarding our data, we observed skewness values of 0.417 (younger) and 0.290 (older) that correspond to a distribution approximatively symmetric (range for normal distribution between -0.5 and 0.5. Finally, we checked if the median value corresponds to the mean value in the two groups, and we did not observe large difference (younger: 23 vs 23.49; older: 67 vs 67.33). For these reasons, we preferred to refer to mean value in the table, and added median value as footnote (**Table 1, Page 13: Lines 5-7**).

**Comment 3.** Although the authors corrected the manuscript according to my other comments, I still have concerns regarding the number of the included participants.

**Response.** We acknowledge that our results would benefit from a larger number of participants. However, as discussed in the first round of revision, it was not possible to include as many participants as in genetic studies due to extensive neuropsychological testing. We hope that adding a sensitivity analysis as requested by Reviewer 2, in addition of explicit statement in the previous version that sample size is a limitation of our study, will remove at least partly your concerns.

**Reviewer #3:**

The paper by Apa and colleagues presents a very interesting investigation on how different genotypes of the COMT gene can influence performance in various tests evaluating executive functions in both young and older individuals. The authors find a generally inferior performance in the older group across the three executive processes and, additionally, lower performance in the inhibition subprocess in older subjects homozygous for valine compared to young individuals with the same genotype. This research is interesting, but it needs improvement in several aspects to be published in Plos One.

**Comment 1.** It would be helpful, for the review process, to include line numbers in the document.

**Response.** This is now done as suggested by Reviewer #3

**Comment 2.** In the method section, information about the type of sociodemographic data collected should be included. Also, in the statistical analysis description section, it would be useful to include how these data will be analyzed.

**Response.** For sociodemographic data, we have now added **Page 6: Lines 13-14** : “Sociocultural level was assessed by asking the number of years completed at school”. We have also added in the statistical analysis description section **Page 12: lines 16-22**: “With regard to demographic data (Sex, education) and global measures of cognition (fluid and crystallized intelligence, speed of processing, Mattis scale), the effect of age was assessed with Student t test between younger and older participants for education level and cognitive performance. Moreover, analyses of variance (ANOVA) were performed separately in younger and older participants to determine effect of COMT polymorphism on these variables in each group. A Pearson chi-square (χ2) test of independence assessed Sex distribution across age groups and, for each age group, across the three genotypes.”

**Comment 3.** In the description of the N-back test, it would be clearer to simply refer to it as the 2-back test.

**Response.** The N-back task we used is now referred a 2-back task across the text (see **Page 8: line 19, Page 12: line 1, Page 19: line 22, Page 22: line 13**)

**Comment 4.** In my opinion, the inclusion of a verbal fluency test as an index of shifting is not fully justified. I believe that a more detailed explanation is needed on how this test relates to that executive subprocess.

Response. As indicated in the previous version of the manuscript, the verbal fluency score was considered as a measure of shifting abilities by Eslinger & Grattan (1993). Indeed, besides assessing access to phonological and semantic information stored in long-term memory, verbal fluency tasks are usually considered as requiring strategic search within phonological and semantic networks. There is nevertheless debate on the importance of contribution of shifting abilities to performance on phonemic and semantic fluency (for example, Nutter-Hupham et al., 2008; Troyer et al., 1997). Consequently, we considered that the best measure to include in the shifting composite score was the sum of words produced for both phonemic and semantic tasks. This is now briefly state **Page 9: line 14-18**: “Verbal fluency performance is usually considered as a measure of shifting abilities (65). Indeed, fluency tasks requires to implement and alternate between several search strategies. However, the contribution of search strategy processes may differ between phonological and semantic tasks (70, 71), and consequently the shifting score used included all verbal fluency tasks”.

70. Troyer, A. K., Moscovitch, M., & Winocur, G. (1997). Clustering and switching as two components of verbal fluency: Evidence from younger and older healthy adults. *Neuropsychology*, [11](https://doi.org/10.1037/0894-4105.11.1.138)([1](https://doi.org/10.1037/0894-4105.11.1.138)), 138–146. doi:10.1037/0894-4105.11.1.138

71. Nutter-Upham, K. E., Saykin, A. J., Rabin, L. A., Roth, R. M., Wishart, H. A., Pare, N., & Flashman, L. A. (2008). Verbal fluency performance in amnestic MCI and older adults with cognitive complaints. *Archives of Clinical Neuropsychology*, [23](https://doi.org/10.1016/j.acn.2008.01.005)([3](https://doi.org/10.1016/j.acn.2008.01.005)), 229–241. doi:10.1016/j.acn.2008.01.005

**Comment 5.** The "Global cognition" section should appear just before the description of the neuropsychological tests used, as it represents an initial evaluation of the subjects.

Response. The section is now as suggested by Reviewer #3 (see **Page 7: line 17**)

**Comment 6.** However, my major concern is about the way the data analyses were performed as they seem redundant at some point. The authors use t-tests (to analyze the results of both groups regardless of genotype) and ANOVAs (to see the effects of group and genotype) and subsequently report group comparisons from t-tests and also the main group effects in the ANOVA. This information is practically the same. In my opinion, it would only be necessary to use the ANOVA to observe these effects. Additionally, I do not quite understand why the authors have "created" 6 groups. Using a 2-way ANOVA can include group (with two levels: young and older) and genotype (three levels: Val/Val, Met/Met, and Met/Val) as factors.

Response. The rationale for our study was first to determine the presence of age-related effects on the three composite scores we created, and next to explore effect of genotypic variations on executive processes that are affected by aging. However, we obtained age-effect on the three scores, and thus the three were included in analyses assessing genetic effects. We have slightly reformulated the end of the introduction to make this point more explicit. **Page 5: Lines 14-17**: “Second, we tested the COMT allelic variance effects on the executive processes for which an age-effect was found. This was done on the six groups of subjects by combining age (younger, older) and genotype (Met/Met, Val/Met, Val/Val).” Regarding your second comment, as indicated **Page 13: Line 3**, we decided to include six groups, and not stratifying by age and genotype in order to enter the tree executive scores in the same analysis, and not perform three separate ANOVAs. This was done to decrease the number of statistical tests and risk of false positive.

**Comment 7.** I believe it would be advisable to perform the Hardy-Weinberg equilibrium test to analyze if the genotype distributions in the sample are comparable to the general population.

**Response**. We chose not to include participants directly from the general population until we reached the set N, which meant that we should have tested whether the frequency distribution of the different genotypes in our sample matched that of the population. Instead, we decided to create groups of roughly equivalent size based on a database available in the context of another research protocol (see Reuter et al., 2005 for a similar recruitment protocol). It therefore seems inappropriate to carry out the Hardy-Weinberg equilibrium test to analyze if the genotype distributions in the sample is comparable to the general population. This is now stated **Page 6 lines 4-7**: “Participants were recruited from a local database at the GIGA-CRC. They were initially enrolled through advertisement in senior centers, universities, and drugstores. Possible subjects offered inclusion criteria presented at the laboratory first for DNA sampling using Isohelix® buccal swabs” and **Page 6 Lines 17-18**: “The selection of participants from an existing database made it possible to form groups of roughly equivalent size for each genotype (see (61) for a similar procedure).”

61. Reuter, M., Peters, K., Schroeter, K., Koebke, W., Lenardon, D., Bloch, B., & Hennig, J. (2005). The influence of the dopaminergic system on cognitive functioning: A molecular genetic approach. Behavioural and Brain research, 164, 93-99. doi:10.1016/j.bbr.2005.06.002

**Comment 8.** Finally, the sample size may be considered small for a genetic study; however, the authors could report statistical power or at least conduct sensitivity analyses to observe possible violations of Type II error.

**Response.** A sensitivity analysis was performed for our ANOVA including the three executive composite scores. Regarding the between effect (Group effect), with a total sample size of 100 and six groups, we were able to detect effect size of *f* = .30 which corresponds to an η^2^ = .08 and is an intermediate effect size (*d* Cohen = 0.60). This is now indicated **Page 13 Lines 13-16.**
